# New qPCR protocol to detect *Diplodia corticola* shows phoretic association with the oak pinhole borer *Platypus cylindrus*

**DOI:** 10.1002/ps.6994

**Published:** 2022-06-04

**Authors:** Emigdio Jordán Muñoz‐Adalia, Andreu Meijer, Carlos Colinas

**Affiliations:** ^1^ Department of Crop and Forest Science University of Lleida Lleida Spain; ^2^ Forest Sciences Center of Catalonia (CTFC) Carretera St. Llorenç de Morunys Solsona Spain

**Keywords:** Botryosphaeria canker, C‐value, *Diplodia* sp. diagnosis, Inoculum detection, Phoresy, Platypodinae

## Abstract

**BACKGROUND:**

Botryosphaeria canker (causal agent: *Diplodia corticola*) is considered one of the most important diseases of cork oak (*Quercus suber*) stands since it necrotizes the subero‐phellodermic layer preventing cork regeneration after harvesting. One of the most intriguing etiological issues of this disease is its plausible spreading through trunk borer insects. In this study, we highlight the phoretic relationship between *D. corticola* and the oak pinhole borer *Platypus cylindrus* (Coleoptera, Curculionidae) which massively colonizes debarked cork oaks in southern Europe. We extracted DNA from 154 adults of *P. cylindrus* collected in six cork oak stands in north‐eastern Spain during the summer of 2021. We developed a new nested quantitative PCR‐based protocol for quick detection of *D. corticola* carried by insects.

**RESULTS:**

The use of real time amplification of a highly specific mitochondrial marker allowed us to detect spore loads down to a single conidium within the first 29 cycles of qPCR. The 29.62% of insect pools (corresponding to 31.16% of studied insects) resulted in an estimated spore load higher than one conidium/insect, with a moderate value of mean spore load for the whole dataset (~33 conidia/insect). Estimated spore load was significantly higher in May and August, regardless of insect abundance in the field.

**CONCLUSION:**

This study provides new tools for diagnosis of this emergent pathogen that would be useful for developing monitoring strategies and epidemiological studies. © 2022 The Authors. *Pest Management Science* published by John Wiley & Sons Ltd on behalf of Society of Chemical Industry.

## Graphical abstract

The study confirms the role of *Platypus cylindrus* as carrier of the emerging pathogen *Diplodia corticola* in European cork oak forests using a highly specific molecular marker.

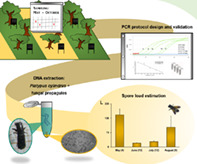

## 1 INTRODUCTION

The ascomycete fungus *Diplodia corticola* Phillips, Alves & Luque (Botryosphaeriaceae) is the causal agent of Botryosphaeria canker, considered as an emergent disease in Mediterranean landscapes where it infects a wide variety of hosts (*e.g., Quercus afares* Pomel, *Quercus canariens*is Willd., *Quercus coccifera* L. and *Quercus ilex* L.) with special incidence in cork oak (*Quercus suber* L.). The fungus is also considered as an exotic pathogen in American oaks (*Quercus agrifolia* Née, *Quercus chrysolepis* Liebm., *Quercus rubra* L. and *Quercus virginiana* Mill.).^1^, ^2^ *Diplodia corticola* causes mortality in seedlings, and severe necrosis, wilting, dieback, and strong reduction in phellem regeneration after cork stripping in mature cork oaks. The latter symptom has a noticeable economic impact since fungal infections cause dramatic reductions in revenues of cork harvesting. Hence, canker occurrence poses a major threat for the viability of traditional and sustainable forest use in the middle term.^3^, ^4^ Despite the relevance of this pathology in Mediterranean landscapes, there are still aspects related to the infection process as well as the predisposing factors that require further research for being elucidated. The inoculum is thought to be spread mainly by wind^5^ and rain drops as described in other species of Botryosphaeriaceae^6^, ^7^ being also possible the presence of *D. corticola* as a latent pathogen in oaks. Insect‐mediated transmission (zoochory) is considered as a possible dissemination path for Botryosphaeria canker as well, but little is known about the association between the pathogen and insects that colonize *Q. suber* stands.

The oak pinhole borer (OPB), *Platypus cylindrus* F. (Coleoptera, Curculionidae, Platypodinae), is among the main causal agents of cork oak decline in Algeria, Morocco, and Portugal, although in Tunisia and Spain it has been traditionally considered as a secondary pest (that is, mainly associated with weakened trees). This ambrosia (xylomicetophagous) beetle usually colonizes stressed or dying hosts by excavating breeding galleries in the trunk, showing preference for debarked trees. When massive infestations occur, this species can kill the tree after 3–18 months, and it can eventually colonize healthy hosts severely reducing the vigor of the stand.^8^

The flight phenology of this insect during the summer completely covers cork harvesting season in Spain and Portugal,^8^, ^9^ this spatial–temporal coincidence suggests its possible participation in the effective spreading of *D. corticola* to healthy hosts. OPB carries pathogenic fungi, for instance, *Ceratocystis platani* (J.M. Walter) Engelbrecht & Harrington which is considered an invasive pathogen of *Platanus* spp.,^10^ other decay‐associated fungi as *Raffaelea* sp. and *Acremonium* sp.,^11^ and the charcoal canker causal agent *Biscogniauxia mediterranea* (De Not.) Kuntze.^12^ On the contrary, there is no confirmation of phoretic association between OPB and *D. corticola* in European ecosystems, despite the confirmed association of OPB with members of Botryosphaeriaceae.^11^ Consequently, the aims of this study were (i) to provide a specific marker‐based molecular method for detecting propagules of *D. corticola* in borer insects, (ii) to investigate whether *D. corticola* is phoretically associated with OPB during its summer dispersion flight, and (iii) to evaluate the spore load carried by this insect in managed cork oak stands in North‐eastern Spain.

## 2 MATERIAL AND METHODS

### 2.1 Field sampling, insect identification, and DNA extraction

Six sampling plots were set in Catalonia (North‐eastern Spain) in managed mature cork oak stands between April and October 2021 (Table 1) for monitoring *P. cylindrus* summer flight period.^13^ We established this study in the county of La Selva (Girona, Spain) in the core area of the NE Spain cork production region. We selected all the mature *Q. suber* stands in the county over 20 ha, that had been harvested in the three previous years. An additional plot (plot 6) located in the Vallès Oriental county was included since previous infestations by OPB had been reported in the area. Two slot‐traps (*i.e*., 500 × 140 × 600 mm black plastic traps supported by two metallic bars. Trap surface has several access slots that let insects drop into a removable drawer with drainage holes to avoid water accumulation; Theysohn, Germany) were set 1.60 m above ground in each plot on 28^th^ April 2021. Traps were located close to recently debarked trees and separated from each other approximately 100 m. Each trap was baited with synthetic pheromone specifically formulated for OPB (Cilindriwit®, Witasek, Germany). Pheromones were replaced according to manufacturer´s instructions without removing old lures from the trap. Relative air humidity and temperature were recorded every hour by an ELUSB‐2 data logger (Lascar Electronics, UK) located between the two traps in each sampling plot.

**Table 1:** Sampled plots during *P. cylindrus* flight period of 2021 in Catalonia

Plot code	County	Coordinates	*Quercus suber* diameter (cm)*	Last cork harvest	Symptomatology of Botryosphaeria canker
1	La Selva	41.850730 N 2.834706 E	31.70 ± 4.48	2020	Symptomatic trees in the stand
2	La Selva	41.926775 N 2.631814 E	34.00 ± 4.09	2020	Symptomatic trees in the stand
3	La Selva	41.879136 N 2.600310 E	19.99 ± 3.26	2020	Symptomatic trees in the stand
4	La Selva	41.829946 N 2.572938 E	35.27 ± 3.51	2020	Symptomatic trees in the stand
5	La Selva	41.902445 N 2.599218 E	38.52 ± 3.49	2020	Symptomatic trees in the stand
6	Vallès Oriental	41.658137 N 2.537927 E	36.16 ± 2.61	2018	No symptomatic trees in the stand

* Diameter was measured in five trees surrounding insect traps (randomly selected). Mean value and standard error are shown.

The traps were surveyed every week between the 4th of May and the 1st of September (13th October in plot 6). All trapped beetles were collected and the sex of OPBs was assessed according to morphological features^8^ under a Leica ZOOM™ 2000 binocular lens (Leica, Germany). After identification, all *P. cylindrus* were stored separately in 1.5 mL sterile tubes at −20° C until being processed.

The complete body of 154 OPBs (*i.e*., 70 ♂♂ and 84 ♀♀; Table 2) was used for DNA extraction without surface sterilization in order not to remove any fungal propagules weakly attached to the exoskeleton. Individuals were randomly selected among captured adults to analyze approximately 10% of total captures each month (minimum per plot for complete study period: 13 insects). OPBs were selected to keep balanced sex ratio in each sampling event, nevertheless females were added when the number of males resulted insufficient (Table 2).

**Table 2:** Number of *P. cylindrus* included in each pool (sampling period: 28^th^ April to 13^th^ October 2021, first trap survey: 4^th^ May)

Plot code	N insects analyzed per sampling period (♂♂/♀♀)					
	May	June	July	August	September	October
1	2/2	1/3	3/3	0/2	Ø	n.a.
2	2/2	5/5	4/4	4/4	1/1	n.a.
3	2/2	2/3	0/3	0/1	Ø	n.a.
4	2/2	5/5	4/4	4/4	1/1	n.a.
5	0/2	3/5	4/5	Ø	Ø	n.a.
6	1/1	4/4	5/5	5/5	4/4	2/2
Total per month	9/11	20/25	20/24	13/16	6/6	2/2
% of total insects	12.99	29.22	28.57	18.83	7.79	2.60

Abbreviations: Ø, Absence of captures; n.a., date out of sampling period.

Selected insects were frozen in liquid nitrogen for 5 s and transferred to sterile 2 mL screw tap tubes containing one sterilized steel ball (diameter: 4 mm). Then samples were ground in a Bead Mill 24 (Fisherbrand, USA) for 20 s at 3.2 m s^−1^. Each tube was briefly centrifugated for 1 min at 11 000 × *g* after disruption to obtain a powder pellet, and then stored at −20° C. Total DNA from each insect was extracted individually using E.Z.N.A.® Plant DNA Kit (Omega‐Bio‐tek Inc., USA) following manufacturer´s instructions. The quality and quantity of extracted DNA was measured using a spectrophotometer (NanoDrop 1000 Thermo Scientific, USA). Extracted DNA was then pooled as described in Table 2 (*i.e*., 27 pools, 5 μL of DNA from each OPB) for further analysis.

### 2.2 Molecular detection of *Diplodia corticola*

A novel quantitative nested PCR protocol was designed for this study. The detection method was based on the amplification of a target region in a highly specific mitochondrial marker. The use of mitochondrial DNA shows advantages such as the higher number of copies of the target marker per cell, as well as the existence of polymorphisms in some cryptic species,^14^ thus being a promising alternative for pathogen surveys in plant material and other environmental samples. Hence, we manually selected mitochondrial genes among those annotated in *D. corticola*´s genome (assembly reference in NCBI database: ASM188384v1) for further analyses. Specifically, we selected mitochondrial DNA helicase gene (NCBI reference: BKCO1_2400083), this gene was not annotated in other *Diplodia* spp., nevertheless the similarity provided by searching using blastn algorithm^15^ [whole‐genome sequencing shotgun contigs database (wgs); *Diplodia* sp. taxid: 66735] showed sequence similarities >86% and query coverages of 99% in other related taxa as *Diplodia sapinea* (Fr.) Fuckel and *Diplodia seriata* De Not. Then, different primer pairs were designed using Primer3web 4.0.0. software (https://primer3.ut.ee/) to amplify candidate target regions. Primers were *in silico* aligned to target gene using Geneious Prime^©^ 2020.1.2. (Biomatters Ltd. New Zealand). The selected sequence to be amplified by the protocol was checked to yield 100% identity and query cover against *D. corticola*´s genome, as well as to anneal neither with the genome of other *Diplodia* spp. nor other fungal genomes using blast (databases: nr/nt and wgs; taxid: 66735 and 4751).

**Figure 1:**
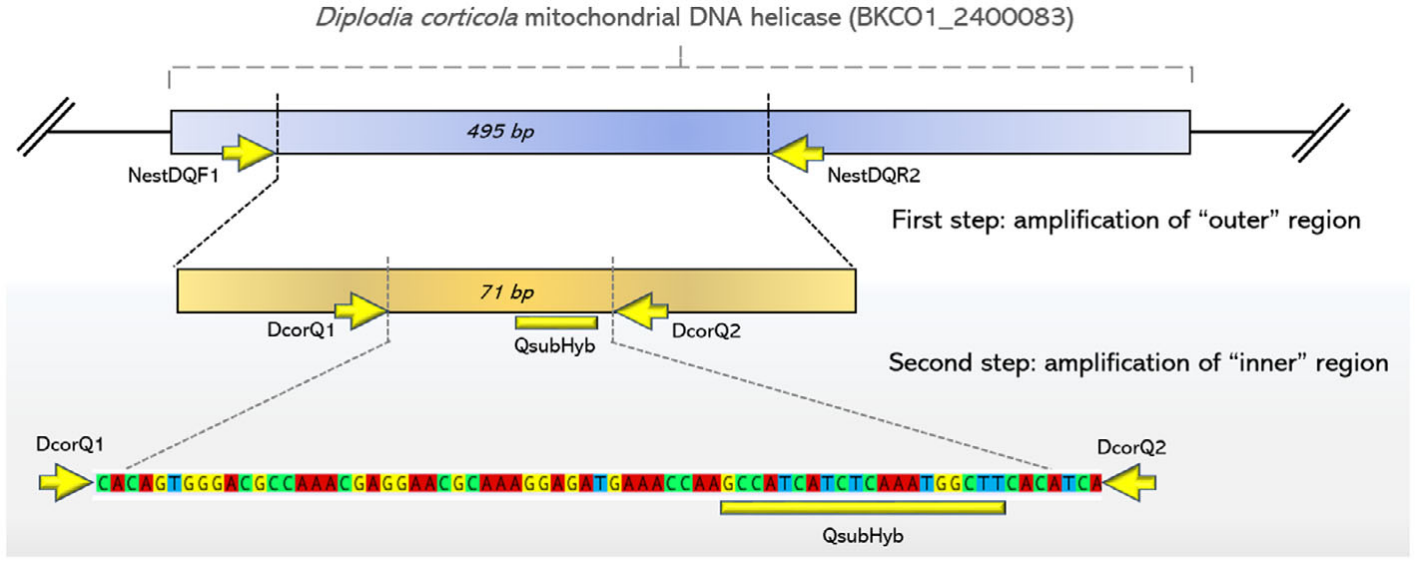
Scheme of nested qPCR protocol designed for detecting *D. corticola*.

The first step of protocol consisted of amplification of a 495 bp amplicon of mitochondrial DNA helicase gene by PCR (Fig. 1). PCR reaction was performed using MyTaq DNA Polymerase (meridian Bioscience Inc., USA) in 50 μL of reaction volume [*i.e*., 1 μL of polymerase, 0.8 μL of each primer (25 μm), 10 μL of 5× buffer, 35.4 μL of double sterilized MiliQ water, and 2 μL of template DNA (pools)] in an Eppendorf Mastercycler nexus X2 (Eppendorf, Germany). A specific primer pair NestDQF1/NestDQR2 (5´‐ACGGTGCATGAGAGACTTGT‐3′/5´‐TGCTTGATTTCCACGGCTTC‐3′) was designed for amplifying this region. PCR protocol consisted of 1 min at 95° C of denaturation, followed by 35 cycles of 15 s at 95° C, 15 s at 54° C, and 10 s at 72°C; the final elongation consisted of 10 min at 72° C. The second step of nested PCR protocol was performed by quantitative real‐time PCR (qPCR) using the primer pair DcorQ1/DcorQ2 (5´‐GATCTGCGAAGCAAGAGGAC‐3′/5′‐GTGGGGAGTGGATTGGAGTA‐3′) as well as a specific probe (QsubHyb: 5´‐GCCATCATCTCAAATGGCTT‐3′; probe modification: 5´‐FAM/3′‐TAMRA). The primer pair was designed to amplify a 71 bp fragment in the mentioned mitochondrial DNA helicase gene (Fig. 1). Quantitative real‐time PCRs were performed in a StepOnePlus real time PCR System (Applied Biosystems, USA) using TaqMan^©^ universal PCR Master Mix (Applied Biosystems, USA). Reaction volume was adjusted to 20 μL per sample including 10 μL of PCR master mix, 0.2 μL of each primer (25 μm) and probe (25 μm) as well as 2 μL of template DNA (PCR product from step 1 diluted 1:10), reaction volume was completed with double‐sterilized MiliQ water. The qPCR conditions consisted of 10 min at 95° C of denaturation, followed by 40 cycles of 15 s at 95°C and 1 min at 55° C. Each sample was amplified three times in the second step (qPCR).

### 2.3 Quantification of *D. corticola*

Concentration of target DNA in the studied pools was estimated computing a standard curve. The curve was fitted by correlating the number of PCR cycles required for the fluorescent signal to overpass the background threshold (*i.e*., ct‐values; qPCR step) of five experimental samples with their corresponding DNA concentrations previously adjusted in laboratory conditions. Specifically, DNA from powdered lyophilized mycelium (~100 mg) from the characterized strain of *D. corticola* CAA007‐1 (NCBI GenBank accession numbers: MW699639 and MW699645), was extracted with E.Z.N.A.® Plant DNA Kit according to manufacturer´s instructions. Serial dilutions of extracted DNA (*i.e*., 1:1, 1:10, 1:100, 1:1000, and 1:10000) were prepared using sterile MiliQ water, and then amplified by nested PCR protocol described above. Resulting ct‐values and DNA concentrations were used for fitting a standard curve in R programming environment.^16^ The resulting equation of the standard curve was used for estimating the relative biomass of fungi in each pool (expressed as pg of target DNA per insect).

Once the concentration of *D. corticola*´s DNA was estimated, we calculated the expected spore load carried by each insect (L) using a reference C‐value (*i.e*., size of haploid nuclear genome). The reference C‐value was calculated from those provided in the Fungal Genome Size Database^17^ as described by Ridgway *et al*.^18^ with minor modifications. Briefly, every Botryosphaeriales taxa available in the database were selected to use their C‐values [*i.e., Botryosphaeria* spp., *Macrophomina phaseolina* (Tassi) Goid., *Neofusicoccum parvum* (Pennycook & Samuels) Crous, Slippers & A.J.L. Phillips, *Phyllosticta* spp., and *Saccharata proteae* (Wakef.) Denman & Crous]. The resulting average C‐value (*i.e*., C‐value_ref_ = 37.57 × 10^−3^ ± 2 × 10^−3^ pg/conidium; mean value and standard error) was used as a reference for estimating the number of conidia in the samples. Possible variations of L by month were evaluated for a subset of data including those pools with expected L higher than one conidium/insect. Since L data did not meet normality assumption, a Kruskal‐Wallis rank sum test was computed in R to evaluate variations of L by month. Dunn´s test was used as post‐hoc analysis using the package ‘rstatix’ in R.^19^

## 3 RESULTS

**Figure 2:**
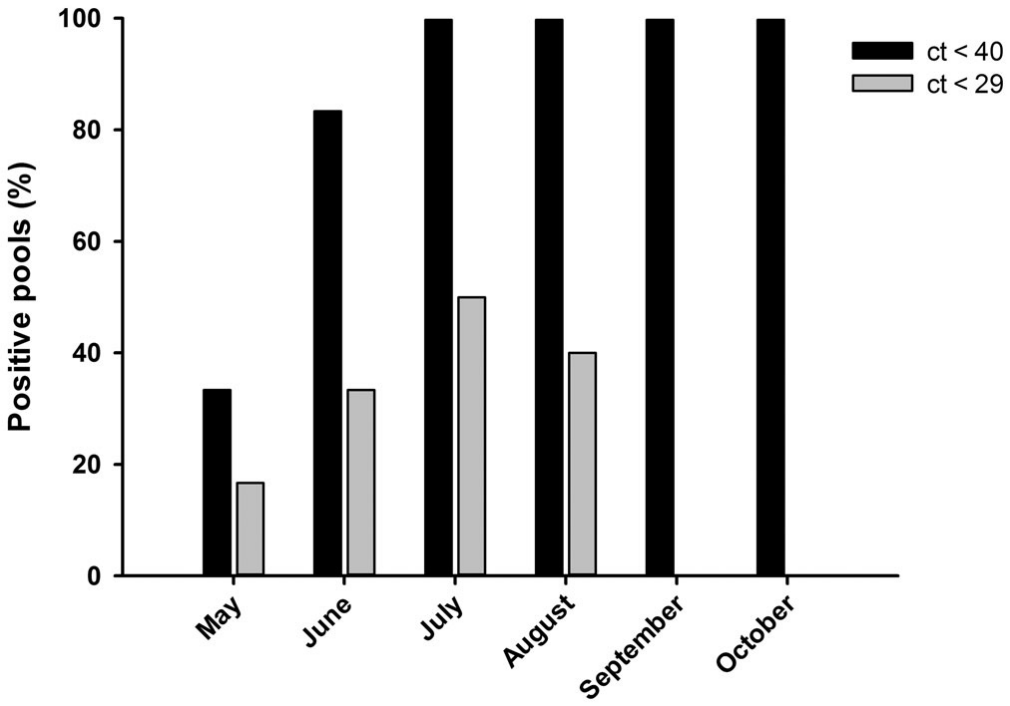
Percentage of pools of *P. cylindrus* with amplification for the specific molecular marker of *D. corticola* by sampling month. Grey bars include pools with at least one replicate with amplification within the 29 first cycles of qPCR.

A total of 154 insects were analyzed in 27 pools of 5.7 ± 0.58 insects/pool for the period comprising May–October 2021 (Table 2). Each pool was amplified by triplicate resulting in 58.08% of positive amplifications in the qPCR step (22 pools amplified at least in one repetition). A total of 8 pools (46.51% of PCR samples; 31.16% of studied insects) showed ct‐values <29, this value corresponded to an expected spore load (L) down to than 1 conidium according to C‐value estimation (see below). Positive amplifications were recorded in pools from all plots thorough the sampling period (Fig. 2), nevertheless insect pools from plot 6 (without symptomatic trees surrounding the traps, Table 1) only provided noticeably delayed amplifications (*i.e*., ct‐values > 37).

**Figure 3:**
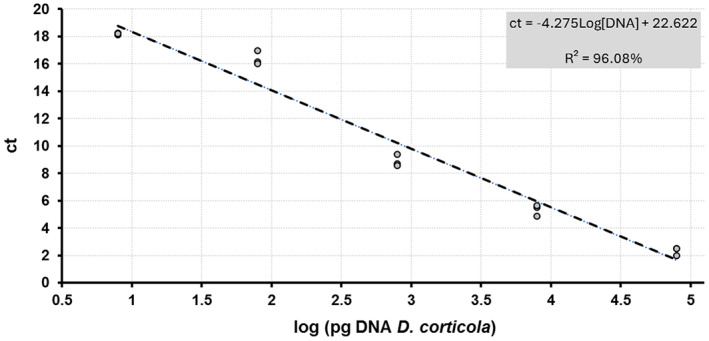
Standard curve fitted for ct‐values and concentration of *D. corticola* DNA from nested qPCR.

**Figure 4:**
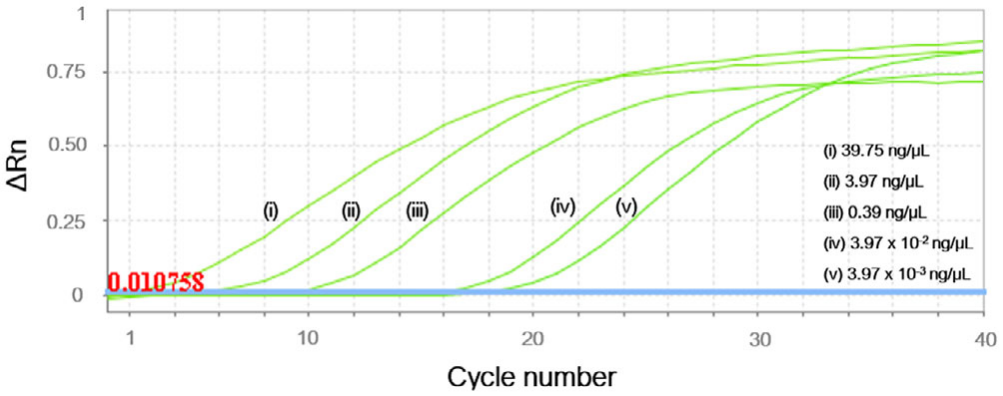
Magnitude of fluorescence signal (ΔRn) obtained with serial dilutions of *D. corticola* DNA positive control. Normalized reporter value of the baseline signal fitted for qPCR run (threshold) is shown as a blue line and noted in red type in the bottom left of the picture.

**Figure 5:**
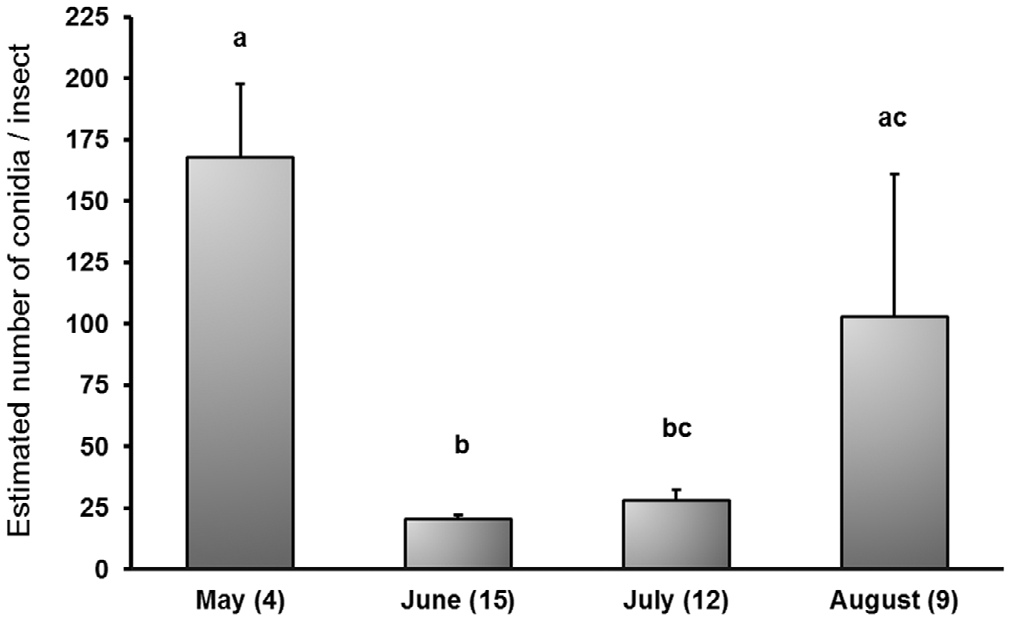
Estimated spore load (L) by month. Only pools with ct‐values <29 were considered. Parentheses indicate the number of *P. cylindrus* in the considered pools. Mean values and standard error are shown. Small letters (a–c) denote significant differences according to Dunn´s test (*P* <0.05).

The linear model computed for serial dilution of DNA extracted from lyophilized mycelium (*i.e*., X axis of Fig. 3, expressed as log_10_) and their corresponding ct‐values (*i.e*., Y axis of Fig. 3; values computed for each qPCR as shown in Fig. 4) significantly fitted the standard curve (R^2^ = 96.08%; *P* <0.01). This model was used for predicting concentrations of DNA in analyzed samples thus providing a mean value of 1.23 ± 0.59 pg target DNA/insect. The values of L per pool were determined using the calculated C‐value_ref_ (37.57 × 10^−3^ ± 2 × 10^−3^ pg/conidium) resulting in an average expected spore load of 32.78 ± 15.87 conidia/insect (complete dataset), 68.46 ± 19.16 conidia/insect (subset: samples with L ≥ 1 conidium/insect) and 58.26 ± 17.14 conidia/insect (subset: samples with L ≥ 1 conidium/pool). The expected spore load significantly varied among the sampling dates (*P*= 0.01). More specifically, L was significantly higher in May and August than in June (*P* ≤0.01 in all cases), while expected spore load did not vary significantly between June and July (*P*= 0.45) (Fig. 5).

## 4 DISCUSSION

In this study, we report for the first time the phoretic association of OPB and the emergent pathogen *D. corticola* in Europe. Both beetle and fungus have overlapping habitats since *D. corticola* colonizes fresh tissue under the cork, and OPB digs through these tissues (*i.e*., phellogen, phelloderm, and phloem) to reach the wood. The occurrence of *Botryosphaeria* species has been reported in adults,^20^ as well as galleries^21^ of *P. cylindrus* pointing the relatively frequent association with this fungal genus. Belhoucine *et al*.^22^ informed of the presence of *D. corticola* both in individuals and galleries of OPB in Algeria. However, that identification was based on a single universal molecular marker (*i.e*., ITS) which is considered as not completely informative for diagnosis of Botryosphaeriaceae,^23^ due to the phylogenetic proximity among *Diplodia* spp. Our study confirms this suggested association using a specific mitochondrial marker, further providing a highly sensitive molecular method for pathogen detection in environmental samples.

Leach^24^ enunciated the four postulates to be fulfilled by insects acting as vectors of plant pathogens. Accordingly, an insect can be considered as a vector if (i) it closely contacts with diseased plants; (ii) it regularly visits healthy plants in suitable conditions for infection development; (iii) the pathogen is present in/on the insect (naturally of after diseased plant visitation); and (iv) the disease develops under controlled conditions after insect visitation. Field observations and previous studies of OPB´s dynamics have demonstrated the postulates i and ii since *P. cylindrus* colonizes both asymptomatic and diseased trees.^8^, ^9^ Besides, here we demonstrated the system *D. corticola* ‐ *P. cylindrus* ‐ *Q. suber* addresses the postulate iii. The latter postulate (iv) needs further research although the plausible presence of the fungus as latent pathogen^25^ could hamper the confirmation of insect boring as unique causative factor of disease development. In consequence, *P. cylindrus* should be considered as carrier (or vector *sensu lato*) of *D. corticola*. Similarly, Pinna *et al*.^26^ found intense phoretic association between *D. corticola* and *Coraebus florentinus* Herbst (Col., Buprestidae) being the fungus present both on the adults’ exoskeleton and in the galleries. On the other hand, Panzavolta *et al*.^27^ also reported phoretic association with *Cerambyx welensii* Küster (Col., Cerambycidae) in declining cork oaks in Italy. *Platypus cylindrus* and *C. florentinus* are able to attack healthy trees and eventually cause new pathogen colonizations.^8^, ^28^ In contrast, *C. welensii* is not expected to promote new infections since it mainly colonizes weakened or almost dead trees, nevertheless Panzavolta *et al*.^27^ suggested oviposition probing habits might facilitate the entry of inoculum in the host. All these considerations point to Botryosphaeria canker disease as a complex pathosystem where subcortical beetles play a relevant role as spreading and wounding agents that cannot be neglected.

The detection method presented here was able to detect DNA concentration below 4 pg μL^−1^ (*i.e*., 7.95 pg/sample) in experimental samples, being able to detect the DNA concentration corresponding to a single conidium within the first 29 cycles of qPCR. In this regard, the number of estimated conidia carried by each individual seems to be moderate (32.78 ± 15.87 conidia/insect). Despite little still being known about the minimum inoculum required for effective colonization of *D. corticola*, it is conceivable that a single spore may be enough to start a new infection. In this regard, previous experimental inoculations with mature mycelium (~1 propagule/cm^2^ in solid culture) resulted in symptoms appearance in mature oaks suggesting that even a rather small number of propagules could be enough for disease development.^4^ In this study, we aimed to detect any association between OPB and *D. corticola*, regardless of whether it occurs randomly or due to the insect's behavior, thereby fungal propagules were not removed from insects’ exoskeleton before samples were processed as reported in other studies.^26^, ^29^ In consequence, it is possible that some of the analyzed insects could have accidentally carried a few spores during their dispersion flight or by contact inside the trap, thus providing a very low amount and/or quality of DNA (*e.g*., 14 pools showed ct‐values >29). Alternatively, these delayed amplification records could have been caused by the presence of *D. corticola* being carried as mycelial debris on the exoskeleton, or as gut content (after accidental or deliberate consumption of mycelium) since *Botryosphaeria* spp. have been isolated from intestinal content of OPB.^12^

*Platypus cylindrus* was loaded with propagules mainly from late spring to the end of summer with relative maxima in May and August (Fig. 5). The estimated spore load did not show any temporal cumulative trend caused by contamination by *D. corticola*´s spores, despite the fact that insect traps could act as passive spore collectors.^30^, ^31^ Temporal pattern described here suggests irregular spore production and/or vegetative growth during the summer with an intriguing lack of propagules in autumn (September onwards), even though mean temperature remained approximately 20 °C and humidity reached 75% in the sampled plots. The apparent absence of inoculum in autumn contrasts with the expected sporulation phenology since other *Diplodia* spp. are known to release conidia after wet periods.^6^, ^7^ On the other hand, in our study area, fungal prevalence was noticeable earlier than reported by Panzavolta *et al*.^27^ who isolated *D. corticola* more frequently from August to October after an intense drought. In addition, the relative maxima of spore load resulted in unpaired OPB dynamics since peaks of emerging adults occurred from early to mid‐June and in mid‐July,^13^ when expected spore load was lower. These observations evidence the lack of knowledge about the lifecycle of the organisms involved in this pathosystem, encouraging development of future studies focused on sporulation ecology and its relationship with carrier insects’ dynamics.

## 5 CONCLUSIONS

In conclusion, we analyzed the unnoticed association between the emergent pathogen *D. corticola* and OPB. The disease is gaining concern for forest owners in Europe, because of the European Union Commission Implementing Regulation (EU) 2020/1498 banning the use of chemical treatments for preventive purposes. The outbreaks of *P. cylindrus* are, in turn, becoming more frequent in Europe and Northern Africa^32^ increasing the need for knowledge about this pathosystem. The detection method provided here will reinforce our capacity to quantify fungal prevalence and it can be implemented to be used routinely in parallel to insects’ surveillances. This information could be of utmost importance in risk assessment studies directed to anticipate damages caused by Botryosphaeria canker.

## CONFLICTS OF INTEREST/COMPETING INTERESTS

The authors declare that they have no conflict of interest.

## AUTHORS CONTRIBUTION

Carlos Colinas and Emigdio Jordán Muñoz‐Adalia conceived the study and developed the methodology. Emigdio Jordán Muñoz‐Adalia coordinated field surveys, set up molecular protocols, and performed the statistical analysis. Emigdio Jordán Muñoz‐Adalia and Andreu Meijer characterized sampling plots and performed laboratory tasks. All authors wrote, reviewed, and approved the final manuscript. The datasets generated during and/or analyzed during the current studyare available in a public repository (https://doi.org/10.34810/data164; https://doi.org/10.34810/data162).

## Data Availability

The data that support the findings of this study are openly available in CORA. Repositorio de Datos de Investigación at https://dataverse.csuc.cat/.
